# Optimization and scale up of microfluidic nanolipomer production method for preclinical and potential clinical trials

**DOI:** 10.1186/s12951-018-0339-0

**Published:** 2018-02-12

**Authors:** Andrew Gdowski, Kaitlyn Johnson, Sunil Shah, Ignacy Gryczynski, Jamboor Vishwanatha, Amalendu Ranjan

**Affiliations:** 1 University of North Texas Health Science Center, 3500 Camp Bowie Blvd, Fort Worth, TX 76107 USA; 2 Tuskegee University, 1200 Montgomery Rd, Tuskegee, AL 36088 USA

**Keywords:** Microfluidic, Optimization, Nanoparticle synthesis, Scale up, Preclinical batch

## Abstract

**Background:**

The process of optimization and fabrication of nanoparticle synthesis for preclinical studies can be challenging and time consuming. Traditional small scale laboratory synthesis techniques suffer from batch to batch variability. Additionally, the parameters used in the original formulation must be re-optimized due to differences in fabrication techniques for clinical production. Several low flow microfluidic synthesis processes have been reported in recent years for developing nanoparticles that are a hybrid between polymeric nanoparticles and liposomes. However, use of high flow microfluidic synthetic techniques has not been described for this type of nanoparticle system, which we will term as nanolipomer. In this manuscript, we describe the successful optimization and functional assessment of nanolipomers fabricated using a microfluidic synthesis method under high flow parameters.

**Results:**

The optimal total flow rate for synthesis of these nanolipomers was found to be 12 ml/min and flow rate ratio 1:1 (organic phase: aqueous phase). The PLGA polymer concentration of 10 mg/ml and a DSPE-PEG lipid concentration of 10% w/v provided optimal size, PDI and stability. Drug loading and encapsulation of a representative hydrophobic small molecule drug, curcumin, was optimized and found that high encapsulation efficiency of 58.8% and drug loading of 4.4% was achieved at 7.5% w/w initial concentration of curcumin/PLGA polymer. The final size and polydispersity index of the optimized nanolipomer was 102.11 nm and 0.126, respectively. Functional assessment of uptake of the nanolipomers in C4-2B prostate cancer cells showed uptake at 1 h and increased uptake at 24 h. The nanolipomer was more effective in the cell viability assay compared to free drug. Finally, assessment of in vivo retention in mice of these nanolipomers revealed retention for up to 2 h and were completely cleared at 24 h.

**Conclusions:**

In this study, we have demonstrated that a nanolipomer formulation can be successfully synthesized and easily scaled up through a high flow microfluidic system with optimal characteristics. The process of developing nanolipomers using this methodology is significant as the same optimized parameters used for small batches could be translated into manufacturing large scale batches for clinical trials through parallel flow systems.

## Background

Many anti-cancer therapeutics are hydrophobic small molecule drugs and must be formulated appropriately so that it will achieve suitable bioavailability when administered systemically [1]. Formulating such nanosystems can be tedious and pose many challenges at both the preclinical and clinical stages of drug development [2] using conventional scale-up processes. The major limitation occurs when transitioning from preclinical formulations to scaling up production for the purposes of clinical trials [3]. This requires a large amount of time and resources. In recent years, microfluidic nanoparticle synthesis strategies have been developed with the goal of providing a successful approach to scale up the nanoparticle synthesis process in a reliable and reproducible manner. Utilizing microfluidics allows for the same parameters used in small scale batches to be applied in parallel during the scale up process [4]. Microfluidics has the possibility to become widely used as the processes are economical, reproducible and amenable to modifications. Further, this can be integrated with many other processes or technologies [5].

Microfluidic devices allow for control over the mixing time by varying solvent flow rates or channel geometry. Moreover, better heat transfer owing to large surface areas enables better temperature control, preventing the formation of large temperature gradients. Finally, as the channel length directly corresponds to the time taken by the reactants to flow through it in continuous flow synthesis, the reaction time can be controlled by tuning the channel length or by adding reagents at precise downstream locations during the particle formation process to quench the reaction [6].

It was our goal in the manuscript to determine if a high flow microfluidic process could be used to synthesize a nanoparticle formulation composed of poly(lactic-*co*-glycolic acid) (PLGA) and lipid surface coating consisting of DSPE-PEG, which we will refer to as a nanolipomer (NLP). Previous attempts have utilized high flow rates for making various formulations of either liposomes or nanoparticles [7–9]. However, hybrid formulations utilizing a core polymeric nanoparticle with a lipid coating for stabilization have only been made at low flow rates [5, 10]. These low flow rates achieve stable and effective nanoparticle formulations but it is uncertain how increasing the flow rate to greater than 1000 times previously reported rates will affect these nanolipomers.

In this system, the herringbone pattern incorporated into the microfluidic channel allows for adequate mixing and coating of the DSPE-PEG onto the polymeric nanoparticle at a high flow rate. The optimization of this formulation proceeded with adjustment of instrument flow settings. Next, formulation parameters were optimized that included polymer concentration, lipid coating concentration, as well as drug concentration. After the nanolipomer was optimized for size, polydispersity, and drug encapsulation parameters, we performed functional assessments that included both in vitro and in vivo studies using a murine model.

## Methods

### Chemicals

PLGA ester-terminated (lactide to glycolide ratio 50:50, molecular weight 45,000–55,000), Nile Red dye, acetonitrile HPLC grade 99.93%, and curcumin were obtained from Sigma-Aldrich (St Louis, MO). 1,2-distearoyl-sn-glycero-3-phosphoethanolamine-*N*-[amino(polyethylene glycol)-2000] (ammonium salt) (DSPE-PEG (2000) Amine (2790.486) (average MW due to polydispersity of PEG)) was purchased from Avanti (Alabaster, Alabama). Roswell Park Memorial Institute (RPMI) 1640 medium, fetal bovine serum, and penicillin/streptomycin were purchased from Hyclone. MilliQ water (Millipore) was used for all experiments.

### Microfluidic production of nanolipomer nanoparticles

The basis of the microfluidic synthesis strategy reported in this manuscript utilizes addition of reagents at two inlet ports. One port with the aqueous phase which contains a lipid/stabilizing component. The second port with the organic phase contains polymer and drug. When these channels combine, a nanoprecipitation reaction occurs and rapid mixing in milliseconds [11] allows for coating the lipid/stabilizing agent onto the polymeric nanoparticle core. Overall, this process allows for high flow rates.

**Fig. 1:**
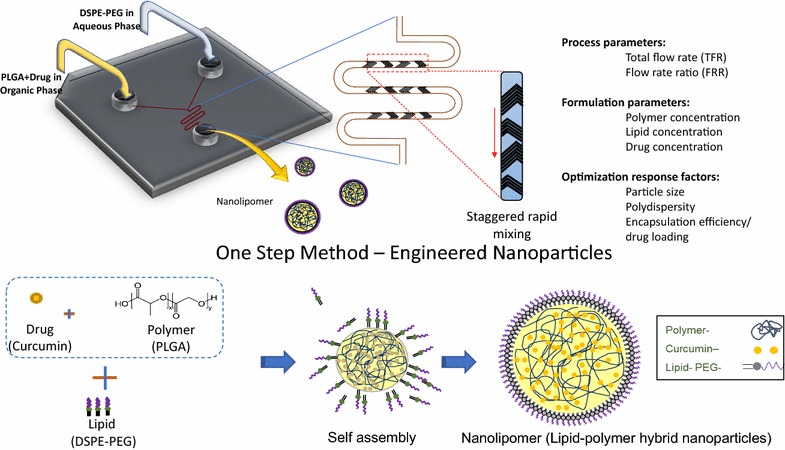
Schematic diagram of synthesis process. Staggered herringbone based microfluidic chip for synthesizing the nanolipomer nanoparticles. The PLGA + drug core is formed by nano-precipitation in the microfluidic chip and introduced from one inlet while the DSPE-PEG lipid coating is injected from the opposite port. The final nanolipomer is collected in the third port after rapid mixing has occurred

All optimization experiments were conducted using the NanoAssemblr Benchtop instrument (manufactured by Precision Nanosystems). PLGA nanoparticles were synthesized by dissolving PLGA ± curcumin in acetonitrile at varying concentrations, this organic solvent mixture was injected in one inlet port in the system. Simultaneously, the DSPE-PEG (at varying concentrations) in 4% ethanol mixture was injected in the other inlet port of the system. Immediately prior to inlet port injection of the DSPE-PEG solution, it was heated in a water bath at 60 °C for 30 s (Fig. 1).

### Instrument parameters optimization

This was achieved by manipulating control of total flow rate (TFR) 2–12 ml/min at PLGA concentrations 5–20 mg/ml and DSPE-PEG concentration of 40% w/w. Next, flow rate ratios of aqueous:solvent (1:1) through (9:1) with 10 mg/ml PLGA in acetonitrile and 40% DSPE-PEG in 4% ethanol while TFR was held at 12 ml/min. NP product was gathered in 15 ml falcon tube while separately disposing the initial volume of 0.25 ml and the final 0.05 ml of NP solution. After the nanolipomer (NLPs) were manufactured, they were processed to remove the aceteonitrile with a solvent exchange method to water in which NLPs were diluted and centrifuged thrice at 1600*g* for 30 min runs in Amicon Ultra Centrifugal Filters 10,000 NMWL. The NLP size and distribution were tested in distilled water and PBS with the Malvern Zetasizer ZS instrument.

### Optimization of NLP formulation parameters

PLGA and DSPE-PEG parameters were optimized with polymer concentration 5, 10, and 20 mg/ml and lipid concentrations of 0, 5, and 10% w/w. This was followed by solvent exchange and determination of size and polydispersity as described above.

After determination of optimized formulation parameters to this point, the encapsulation of curcumin was performed. Curcumin was dissolved in 10 mg/ml of PLGA in acetonitrile so initial concentration of curcumin was varied between 0 and 7.5% curcumin w/w polymer. 10% DSPE-PEG w/w of lipid to polymer ratio was used with a TFR of 12 ml/min and a flow rate ratio (FRR) of 1:1 (aqueous channel input to organic channel input). Solvent exchange to remove non-encapsulated curcumin was performed and the amount of encapsulated curcumin was quantified by UV–Vis plate reader against a standard curve of curcumin in acetonitrile using absorbance at wavelength of 450 nm.

Drug loading and encapsulation efficiency were determined for the different initial concentrations of curcumin. Encapsulation efficiency (EE) was calculated using the following equation: EE = (actual amount of drug encapsulated in nanoparticles)/(starting amount of drug used in nanoparticles) × 100%. Drug loading (DL) was calculated with the equation: DL = (weight of drug in nanoparticles)/(weight of nanoparticles) × 100%.

NLP stability was assessed by incubating 100 μl of 10 mg/ml NLP formulation into 1 ml of molecular biology reagent water (Sigma-Aldrich). Nanoparticles were stored at 4 °C then size and PDI were measured daily for a period of 7 days.

### Fluorescent characterization studies

#### Time-resolved measurements

Fluorescence lifetime and anisotropy decay were measured using FluoTime200 (PicoQuant, GmbH, Berlin, Germany) time domain fluorometer. This instrument, equipped with microchannel plate detector (Hamamatsu, Japan) and a 470 nm pulsed picosecond laser diode provided resolution of 4 ps/channel. The fluorescence lifetime of curcumin-loaded NP was measured at magic angle conditions and data were analyzed with a FluoFit version 5.0 software (PicoQuant, GmbH, Berlin, Germany). The lifetime data were fitted to the multi-exponential deconvolution model:

$$I\left( t \right) = \int_{ - \infty }^{t} {IRF\left( {t^{\prime}} \right)} \mathop \sum \limits_{i} \alpha_{i} e^{{\frac{{ - t - t^{\prime}}}{{\tau_{i} }}}}$$

where *IRF*(*t*^′^) represents the instrument response function at time $t^{\prime}$, *τ*_*i*_ is the lifetime of the ith component, and *α*_*i*_ is the amplitude of decay of the ith component at time t. The average values were calculated as:

$$\bar{\tau } = \sum\limits_{i} {f_{i} \tau_{i} } \quad f_{i} = \frac{{\alpha_{i} \tau_{i} }}{{\mathop \sum \nolimits_{i} \alpha_{i} \tau_{i} }}$$

The anisotropy decays were measured using VV and VH polarizer orientation on the emission side with a 470 nm laser diode. Anisotropy decays were analyzed with multi-exponential fitting model in FluoFit3 program from PicoQuant, Inc (Germany) using following equation.

$$r\left( t \right) = \sum\limits_{i} {r_{i} e^{{ - t/\Phi _{i} }} }$$

#### Steady-state measurements

All measurements were performed in a 1 cm × 1 cm quartz cuvette at room temperature (20 °C). On account of poor water solubility, Curcumin dissolved in 100% ethanol was used as a reference. The nanoparticles were easily dissolved in water. Absorption spectra were collected on a Cary 50 Bio UV–visible spectrophotometer (Varian Inc., Australia). The absorption was scanned from 300 to 500 nm and water was used as a baseline reference. Emission spectra were measured using Cary Eclipse spectrofluorometer (Varian Inc., Australia). The samples were excited at 375 nm, and the emission scanned from 450 to 700 nm in a square geometry set-up.

### Cell viability assay

Prostate cancer cell line C4-2B-luciferase was used to assess cell viability after treatment with NLP or free curcumin. C4-2B-luciferase cells were generously provided by Dr. Even Keller (University of Michigan). Cells were grown in RPMI-Media supplemented with 10% FBS and 1% antibiotic–antimycotic. Upon reaching 70% confluency, cells were subcultured and plated on 96 well flat bottom plates at a seeding density of 2000 cells per well in quadruplicate with the same culture conditions as described above. Cells were allowed to attach for 24 h, media was replaced with fresh media and then cells were treated with NLPs or free drug for a period of either 48, or 72 h with concentration range 0–20 μM curcumin or equivalent dose of NLP. At respective time points, 10 μl Tiazolyl Blue Tetrazolium Bromide (MTT) suspended in PBS at a concentration of 10.5 mg/ml was added to the 200 μl media in each 96 well plate. Cells were incubated with MTT reagent for 3 h, media was then removed and 150 μl of DMSO was added to all wells and mixed. Absorbance was read on BioTek Synergy two Multi-Mode Plate Reader at 570 nm.

### Cellular uptake studies

C4-2B-luciferase cells were plated on glass cover slips in 6 well plates at a density of 0.5 × 10^6^ cells per well and attachment allowed for 24 h. During NLP synthesis step, Nile Red dye was incorporated into PLGA core and excess dye washed out of the formulation. NLP was added to cell culture media at a concentration of 200 μg/ml for 0–6 h time points and at appropriate time washed three times with PBS to remove NLP that was not taken up by cells. Cells were fixed with 4% paraformaldehyde for 10 min, washed, then mounted on slides with Prolong Gold antifade reagent with DAPI. Imaging was performed with the Zeiss LSM 510 confocal microscope.

### NLP retention study

To determine in vivo retention time of NLP, Nile red was incorporated into the NLP formulation as described above. Male nude mice aged 5 weeks were injected via tail vein with 2 mg of final NLP formulation. Fluorescent signal after injection was monitored between 25 min and 24 h using the IVIS animal imaging system (Perkin Elmer, USA). All institutional IACUC animal protocols were followed.

## Results

### Self-assembly of nanolipomers by microfluidic synthesis

**Fig. 2:**
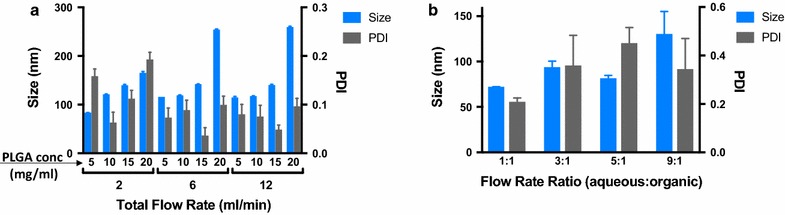
Instrument parameters optimization. **a** Effect of total flow rate (TFR) on the size and polydispersity (PDI) of the nanolipomers at polymer concentration of 5, 10, 15, and 20 mg/ml (DSPE-PEG concentration = 40%, aqueous:solvent FRR = 1:1). **b** Effect of aqueous:solvent flow rate ratio (FRR) on the size and polydispersity (PDI) of nanolipomers at polymer concentration of 10 mg/ml (DSPE-PEG concentration = 40%, TFR = 12 ml/min). (n = 3: mean ± SD)

The instrument parameters were focused on first. Analysis of the effects of adjusting the total flow rate (TFR) showed that increasing TFR impacted the size of the NLPs, particularly at the low (5 mg/ml) and high (20 mg/ml) initial polymer concentrations showed that as the TFR was increased from 2 to 6 ml/min there was a significant increase in size of the NLP. Further, increase in TFR from 6 to 12 ml/min does not have a significant difference in the size of the NLPs. We found minimal change in the nanoparticle size and concentration at the midrange initial polymer range (10 and 15 mg/ml) across the different total flow groups. As expected, we found that in each flow rate group the size of the nanoparticles increased as the PLGA concentration increased. Since the 10 mg/ml concentration of PLGA nanoparticles were stable with low size and PDI at a total flow rate of 12 ml/min, we selected this as the optimal total flow rate (Fig. 2).

Next, the flow rate ratio (FRR) of aqueous solution to organic solvent was determined. The FRR of 1:1 aqueous solution to organic solvent created uniform and small nanoparticles with average size 72 nm and PDI of 0.209. The higher flow rate ratios that were tested yielded NLPs larger in size with a higher PDI (Fig. 2).

**Fig. 3:**
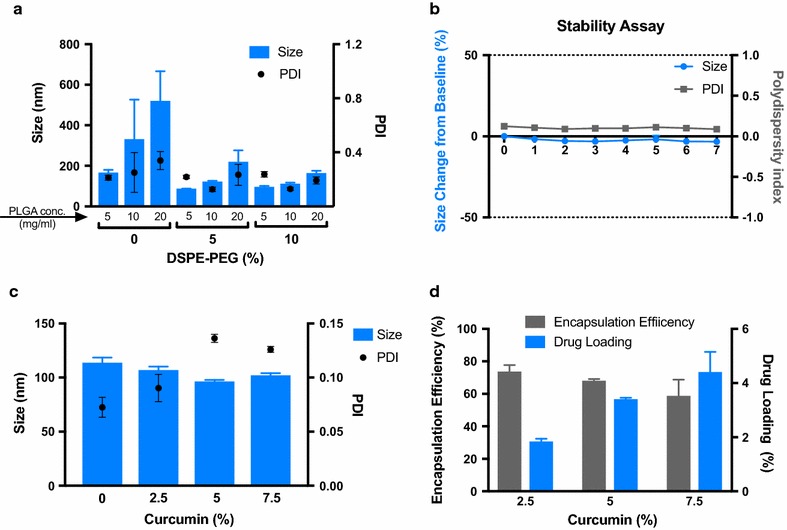
Nanolipomer formulation parameters. **a** Effect of PLGA and DSPE-PEG concentration on the size and PDI of nanolipomers. **b** Nanolipomer stability assay at 4 °C over 1 week. **c** Effect of initial curcumin concentration on size and PDI. **d** Curcumin encapsulation efficiency and drug loading at various initial concentrations of curcumin. (n = 3: mean ± SD)

After the instrument parameters were optimized, attention was turned to optimizing the initial polymer concentrations and lipid coating. Polymer concentration consisting of 5, 10, 20 mg/ml in acetonitrile was tested against an initial concentration of DSPE-PEG at 0, 5, and 10% w/w. When no DSPE-PEG is used (0%) for stabilizing the NLPs, the size is increased, likely due to aggregation. As the DSPE-PEG is introduced at 5 and 10% the particle size stabilizes and size remains small. The 10% DSPE-PEG and 10 mg/ml achieved combination of small nanoparticle size (mean = 112.2 nm, SD 5.24) with low PDI (mean = 0.129, SD 0.01) (Fig. 3). These values were used for the final parameter before the drug encapsulation optimization.

NLPs showed stability when stored at 4 °C over a period of 7 days. There was minimal fluctuation of both size and polydispersity of this formulation (Fig. 3).

### Efficient drug loading using microfluidic synthesis

The size of the NLP was stable as the amount of curcumin used in the initial concentration was increased from 0 to 7.5%. The PDI slightly increased at the 5% (0.136) and 7.5% (0.126) curcumin concentrations compared to the 0% (0.073) and 2.5% (0.090). The encapsulation efficiency at 2.5% initial curcumin was approximately 73.68% and it decreased in a linear fashion to approximately 58.79% at the highest concentration of initial curcumin which was 7.5% w/w. Drug loading peaked at the highest concentration of initial curcumin (7.5%) for this method at 4.41% (Fig. 3).

### NLP fluorescent characterization

**Fig. 4:**
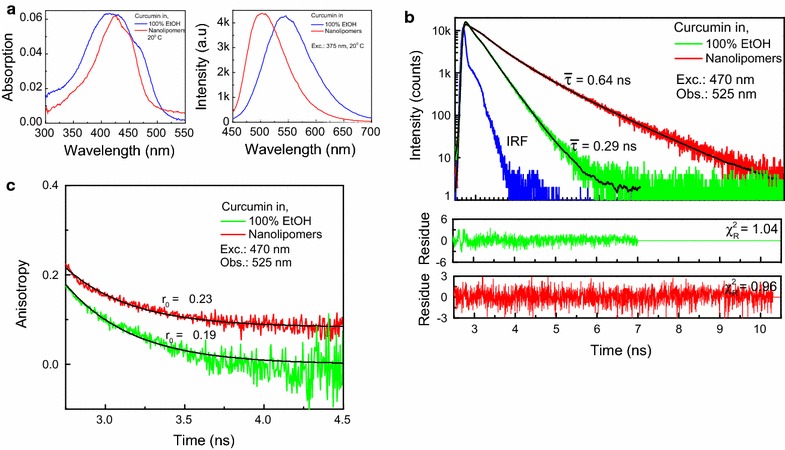
Fluorescence measurements of nanolipomers. **a** Absorption spectrum of curcumin and nanolipomers after curcumin encapsulation. **b** Time resolved lifetime measurements of curcumin vs nanolipomers. **c** Time resolved anisotropy measurements of curcumin vs nanolipomers

The absorption spectra of curcumin and loaded nanolipomers have similar peaks at 424 nm. The NLP absorption spectrum is more structured and narrow after correction for scattering. For steady state emission, the NLPs exhibit a blue shift of approximately 40 nm compared to free curcumin. Time resolved lifetime measurements show that the lifetime of curcumin nearly doubles from 0.29 to 0.64 ns when packed within NLPs. Time resolved anisotropy show a higher anisotropy of 0.23 ns for NLPs as compared to 0.18 ns for curcumin alone. This could be on account of a more rigid environment in NLPs due to packing of curcumin molecules (Fig. 4).

### Nanolipomers are effective in in vitro functional assays

**Fig. 5:**
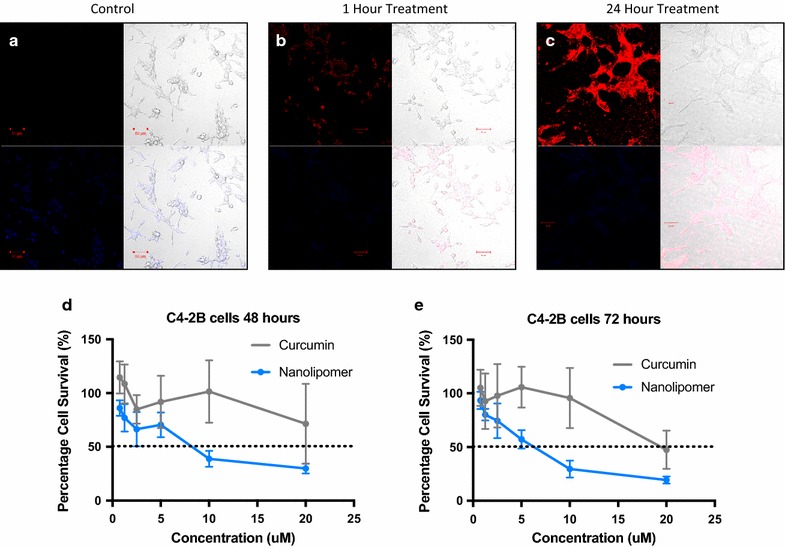
In vitro functional assessment of nanolipomers. **a**–**c**. C4-2B prostate cancer cell uptake experiment at 1 and 24 h. **d** Cell viability of C4-2B cells after 48 h of treatment. **e** Cell viability of C4-2B cells after 72 h of treatment (n = 3: mean ± SD)

Hybrid nanoparticles were verified to have substantial uptake in the C4-2B prostate cancer cell line. The uptake was followed at two time points (1 and 24 h) and shown to have increased uptake at 24 h time point as measured through confocal microscopy (Fig. 5).

In the MTT cell viability assay, the synthesized NLPs were more effective at treating C4-2B cells than the free curcumin at equivalent concentration. This increased effectiveness was true at both the 48 and 72 h time points. The concentration at which the NLPs inhibited 50% of cell viability was considered the IC_50_ of the NLPs. At 48 h the IC_50_ was ~ 8.45 μM while the free curcumin at the 48-h time point didn’t achieve an IC_50_ at the highest concentration tested. At 72 h the IC_50_ of the NLPs was found to be more effective at 6.5 μM, while the IC_50_ of the curcumin was 19.89 μM (Fig. 5).

### NLP coating allows for adequate in vivo retention

**Fig. 6:**
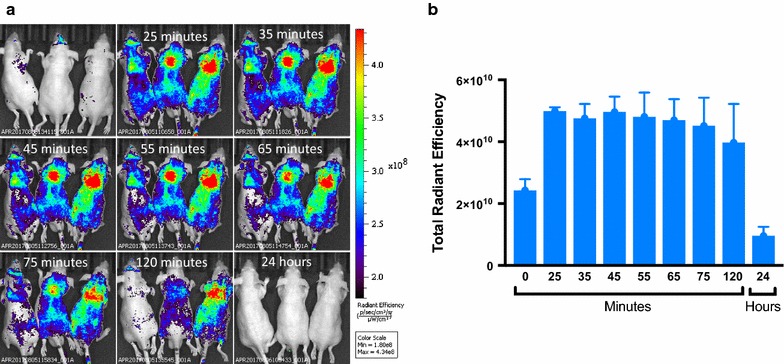
In vivo nanolipomer retention assay. **a** Live animal imaging at various time points after tail vein injection of fluorescently labeled nanolipomer. **b** Quantification of fluorescent signal at various time points. (n = 3: mean ± SD)

NLPs were assessed for their retention in a murine system. We found that the NLPs exhibited relatively stable fluorescent signal during the period from 25 min to 2 h after tail vein injection. At 24 h, both the NLP nanoparticles had been completed removed from the system (Fig. 6).

## Discussion

In this paper, we have demonstrated the ease and scalability of utilizing a microfluidics system to synthesize nanolipomers. Others have reported the use of microfluidic systems for fabrication of nanoparticles and liposomes with various stabilizer coatings at high flow rates [7–9, 12]. However, it has not been demonstrated whether using high flow rates could achieve stable and adequate nanoparticle coating of DSPE-PEG onto PLGA nanoparticles. Utilizing this hybrid concept of PLGA polymeric nanoparticles with a DSPE-PEG lipid coating allows us to combine the advantageous properties of both PLGA nanoparticles and DSPE-PEG [13]. The polymeric nanoparticle provides stable controlled release drug kinetics [14]. The addition of DSPE-PEG allows for decreased protein absorption on the surface of the nanoparticle and a longer circulation time [13].

The optimization strategy that was employed for this project proceeded in an orderly stepwise fashion focusing on two principle components. The parameters intrinsic to the instrument itself were the first components and the variables within the formulation were the second components. The instrument parameters included the total flow rate and flow rate ratio. We based our optimization on the highly desirable nanoparticle product characteristics, small size and low polydispersity. To this point, we found that when total flow rate was increased from 2 to 12 ml/min, there was little change in the NLP size or polydispersity at the polymer concentration in the mid-range that was tested (10 and 15 mg/ml). However, there was an increase in size between the flow rates at the lowest concentration of polymer used (5 mg/ml) and the highest concentration of polymer used (20 mg/ml). The overarching goal of this project was to maximize NLP production while maintaining acceptable characteristics so the total flow rate of 12 ml/min was chosen for the remaining experiments. The microfluidic synthesis process and coating with DSPE-PEG onto polymeric nanoparticles has previously only been reported successful at low flow rates on the order of μl/min [5, 10]. To reduce production times, higher flow rates must be tested and validated for these types of hybrid nanoparticles.

The next aspect of the instrument to be optimized was the flow rate ratio of aqueous phase to organic phase. When increasing this ratio, there was an increase in both size and PDI of the NLP. The shift in the polarity of the solution when rapidly mixing the aqueous and organic phases together results in a supersaturation of hydrophobic molecules in a new solvent and thus causes the precipitation resulting in formation of the PLGA nanoparticle with a small size [15]. However, the PLGA nanoparticles must be stabilized with DSPE-PEG to prevent the nanoparticles from aggregating once they are formed. The change in polarity felt by the amphiphilic DSPE-PEG is less and this lower driving force of the DSPE-PEG to arrange on the hydrophobic PLGA core results in larger core nanoparticles as the flow rate ratio increased. We found the best balance between these two factors to be at the 1:1 flow rate ratio.

The next phase was to optimize the formulation parameters. The three parameters to be optimized were the concentrations of: DSPE-PEG, PLGA polymer, and initial drug used. These parameters could be rapidly optimized due to the ease of manufacturing with the microfluidic system. It was found that as the concentration of PLGA was increased, the nanoparticle size increased. This is likely due to the increase in viscosity of the organic phase that occurs with the increased concentration of polymer. This increased viscosity lowers the diffusion speed of the polymer while mixing and results in larger particles. In addition, as the DSPE-PEG concentration was increased from 5 to 10% we found slight benefits in terms of size and PDI and also found that the particles were most stable in phosphate buffered saline at the 10% concentration (data not shown). Thus DSPE-PEG concentration of 10% was chosen. Further, these particles were stable over the course of 7 days with minimal fluctuation in size and PDI when suspended in water.

In this process, curcumin was chosen as a representative drug for encapsulation within the nanolipomer. Curcumin is hydrophobic and thus serves as an illustrative molecule for many anti-cancer agents [16]. In addition, curcumin is fluorescent so determining its encapsulation within the nanolipomer is also relatively simple [17]. We found this process resulted in a high encapsulation efficiency (58.8%) and drug loading (4.4%) when the initial concentration of curcumin was set at 7.5% w/w polymer. Time resolved lifetime measurements show that the lifetime of curcumin nearly doubles when packed within nanoparticles. This is important in the context of imaging and drug tracking as fluorophores with shorter fluorescent lifetimes are characteristic of weak emitters. By increasing the lifetime and anisotropy of the molecule after encapsulation in nanolipomers, it reconfirms that the curcumin is encapsulated within the nanoparticle and also improves the ability to track the curcumin during in vitro and in vivo experimentation [18].

The DSPE-PEG lipid coating of these nanolipomers has been reported to allow for a more biocompatible formulation and improved cancer cell uptake [19]. We found that these NLPs had increased at 24 h compared to 1 h of treatment. This increased uptake over a longer course of time was evident in the results of the MTT cell viability assay. It was found that treatment with the NLP in prostate cancer cell line C4-2B had a lower IC_50_ value at the 72 h time point than the 48 h time point, indicating improved efficacy at 72 h. Additionally, the NLP was more effective at reducing the prostate cancer cell viability at both the 48 and 72 h times points compared to the equivalent dose of free curcumin.

The NLP formulation was fluorescently labeled and administered in a mouse model to determine the length of time these NLPs were retained after systemic injection. It was found that the NLP was retained in the system and provided a stable signal for up to 2 h after injection. At the 24-h time point after injection the NLP had been cleared.

At the flow rates used in this project, we have estimated the amount of nanopliomer product that could be fabricated. We calculated that it was feasible for one person completing the nanolipomer synthesis, as described, could easily make 14.4 g of product at the flow rate of 12 ml/min during 1 day.

Based on an initial concentration of PLGA at 10 mg/ml and total flow rates ranging from 2 to 12 ml/min, the estimated production rate of product was calculated based on the feasibility for a single operator during 1 day (Table 1).

**Table 1:** NLP production estimate

Polymer concentration (mg/ml)	10 mg/ml	10 mg/ml	10 mg/ml
Total flow rate (ml/min)	2	6	12
Production rate (g/day)	2.4	7.2	14.4

## Conclusion

In this study, we have demonstrated a successful one-step microfluidic process for facilitating the manufacturing and scaling up of nanolipomers. Prior attempts at producing these nanolipomers were burdened by low flow rates, low batch yield, inconsistencies between batches, and difficultly scaling the manufacturing process to make amounts that could be used on the clinical scale. This method provides a simple way to scale up optimization of small batch production techniques that can be used to translate into larger quantities while retaining the same beneficial properties that make nanolipomer nanoparticles advantageous. The successful scaling up of nanoparticle systems using microfluidics can lead to faster clinical approval possibilities thereby addressing the major limitation that hinders successful translation of these nanotherapeutics to reach patients.

## Abbreviations

NLP: nanolipomer
PLGA: poly(lactic-*co*-glycolic) acid
NPs: nanoparticles
TFR: total flow rate
FRR: flow rate ratio
PDI: polydispersity index

## References

[1] Vrignaud S, Benoit JP, Saulnier P (2011). Strategies for the nanoencapsulation of hydrophilic molecules in polymer-based nanoparticles. Biomaterials.

[2] Desai N (2012). Challenges in development of nanoparticle-based therapeutics. AAPS J.

[3] Paliwal R, Babu RJ, Palakurthi S (2014). Nanomedicine scale-up technologies: feasibilities and challenges. AAPS Pharm Sci Techol.

[4] Garg S, Heuck G, Ip S, Ramsay E (2016). Microfluidics: a transformational tool for nanomedicine development and production. J Drug Target.

[5] Valencia PM, Pridgen EM, Rhee M, Langer R, Farokhzad OC, Karnik R (2013). Microfluidic platform for combinatorial synthesis and optimization of targeted nanoparticles for cancer therapy. ACS Nano.

[6] DeMello AJ (2006). Control and detection of chemical reactions in microfluidic systems. Nature.

[7] Correia MG, Briuglia ML, Niosi F, Lamprou DA (2017). Microfluidic manufacturing of phospholipid nanoparticles: Stability, encapsulation efficacy, and drug release. Int J Pharm.

[8] Yanagi T, Tachikawa K, Wilkie-Grantham R, Hishiki A, Nagai K, Toyonaga E, Chivukula P, Matsuzawa S (2016). Lipid nanoparticle-mediated siRNA transfer against PCTAIRE1/PCTK1/Cdk16 inhibits in vivo cancer growth. Mol Ther Nucleic Acids.

[9] Li B, Luo X, Deng B, Wang J, McComb DW, Shi Y, Gaensler KM, Tan X, Dunn AL, Kerlin BA (2015). An orthogonal array optimization of lipid-like nanoparticles for mRNA delivery in vivo. Nano Lett.

[10] Zhang L, Feng Q, Wang J, Zhang S, Ding B, Wei Y, Dong M, Ryu JY, Yoon TY, Shi X (2015). Microfluidic synthesis of hybrid nanoparticles with controlled lipid layers: understanding flexibility-regulated cell-nanoparticle interaction. ACS Nano.

[11] Zhigaltsev IV, Belliveau N, Hafez I, Leung AK, Huft J, Hansen C, Cullis PR (2012). Bottom-up design and synthesis of limit size lipid nanoparticle systems with aqueous and triglyceride cores using millisecond microfluidic mixing. Langmuir ACS J Surf Colloids.

[12] Karnik R, Gu F, Basto P, Cannizzaro C, Dean L, Kyei-Manu W, Langer R, Farokhzad OC (2008). Microfluidic platform for controlled synthesis of polymeric nanoparticles. Nano Lett.

[13] Zhang L, Chan JM, Gu FX, Rhee JW, Wang AZ, Radovic-Moreno AF, Alexis F, Langer R, Farokhzad OC (2008). Self-assembled lipid—polymer hybrid nanoparticles: a robust drug delivery platform. ACS Nano.

[14] Grottkau BE, Cai X, Wang J, Yang X, Lin Y (2013). Polymeric nanoparticles for a drug delivery system. Curr Drug Metab.

[15] Belliveau NM, Huft J, Lin PJ, Chen S, Leung AK, Leaver TJ, Wild AW, Lee JB, Taylor RJ, Tam YK (2012). Microfluidic synthesis of highly potent limit-size lipid nanoparticles for in vivo delivery of siRNA. Mol Ther Nucleic Acids.

[16] Ranjan AP, Mukerjee A, Gdowski A, Helson L, Bouchard A, Majeed M, Vishwanatha JK (2016). Curcumin-ER prolonged subcutaneous delivery for the treatment of non-small cell lung cancer. J Biomed Nanotechnol.

[17] Chignell CF, Bilski P, Reszka KJ, Motten AG, Sik RH, Dahl TA (1994). Spectral and photochemical properties of curcumin. Photochem Photobiol.

[18] Berezin MY, Achilefu S (2010). Fluorescence lifetime measurements and biological imaging. Chem Rev.

[19] Bose RJ, Lee SH, Park H (2016). Lipid-based surface engineering of PLGA nanoparticles for drug and gene delivery applications. Biomater Res.

